# Supplementary material for: Sources of Dietary Salt in North and South India Estimated from 24 Hour Dietary Recall
Source: Nutrients. 2019 Feb 1;11(2):318. doi: 10.3390/nu11020318 (PMC6412427; doi:10.3390/nu11020318)
Supplement: Supplementary file 1 [file nutrients-11-00318-s001.pdf]

Supplementary file

Table S1: Mean percent salt contribution by sex

Food group	Andhra Pradesh			Delhi and Haryana		
	**Male (n=380)**	**Female (n=377)**	**p-value**	**Male (n=250)**	**Female (n=276)**	**p-value**
**Added salt**	**87.21** (85.19 to 89.24)	**88.22** (87.14 to 89.31)	0.780	**82.90** (80.59 to 85.21)	**84.07** (81.89 to 86.25)	0.943
**Mean percent sodium contribution (without added salt)**						
**Beverage (alcoholic)**	**0.40** (0.16 to 0.65)	**0.00** (0.00 to 0.00)	0.004	**0.12** (0.05 to 0.19)	**0.02** (0.00 to 0.05)	0.022
**Beverage (non-alcoholic)**	**0.26** (0.04 to 0.47)	**0.08** (0.00 to 0.18)	0.269	**2.12** (0.21 to 4.03)	**0.49** (0.15 to 0.83)	0.205
**Bread and bakery products**	**0.99** (0.00 to 2.37)	**1.95** (0.07 to 3.82)	0.842	**14.87** (10.17 to 19.57)	**11.16** (7.07 to 15.26)	0.491
**Cereal, grains and products**	**4.34** (2.99 to 5.68)	**4.59** (3.94 to 5.23)	1.000	**4.92** (3.40 to 6.45)	**6.56** (2.90 to 10.21)	0.840
**Dairy and dairy products**	**34.58** (28.39 to 40.76)	**38.77** (32.68 to 44.85)	0.691	**45.45** (38.54 to 52.36)	**50.93** (42.97 to 58.88)	0.620
**Fats and edible oils**	**0.01** (0.00 to 0.03)	**0.03** (0.00 to 0.08)	1.000	**1.33** (0.40 to 2.26)	**0.88** (0.00 to 2.07)	1.000
**Fish and seafood**	**7.62** (4.51 to 10.72)	**6.82** (2.37 to 11.28)	1.000	**0.16** (0.00 to 0.44)	**0.01** (0.00 to 0.03)	0.607
**Fruits and vegetables**	**16.53** (12.59 to 20.47)	**17.08** (13.63 to 20.54)	1.000	**17.53** (14.51 to 20.54)	**19.59** (17.07 to 22.12)	0.611
**Meat, poultry and eggs**	**34.84** (25.92 to 43.75)	**30.20** (24.12 to 36.29)	0.803	**5.28** (1.59 to 8.96)	**3.63** (1.38 to 5.88)	0.911
**Snack foods**	**0.18** (0.00 to 0.56)	**0.10** (0.00 to 0.29)	1.000	**8.09** (5.40 to 10.78)	**6.72** (3.21 to 10.23)	1.000
**Sugar, honey and related products**	**0.26** (0.19 to 0.33)	**0.39** (0.27 to 0.50)	0.152	**0.14** (0.00 to 0.30)	**0.02** (0.00 to 0.05)	0.369

Note: The analysis is based on regression (compatible with svy command). Post-estimation *contrast* command was used to compare sex in each state and sex by state. The analysis is adjusted for multiple comparisons using Bonferroni’s method.

Table S2: Mean percent salt contribution by age group

Food group	Andhra Pradesh				Delhi and Haryana			
	20-39y (n=247)	40-59y (n=256)	60y up (n=254)	p-value	20-39y (n=193)	40-59y (n=180)	60y up (n=153)	p-value
**Added salt**	**88.04** (86.08 to 90.00)	**87.29** (85.50 to 89.09)	**87.20** (86.06 to 88.33)	1.000<br>1.000<br>1.000	**83.50** (81.17 to 85.83)	**82.68** (79.66 to 85.70)	**84.99** (83.05 to 86.93)	1.000<br>1.000<br>1.000
**Mean percent sodium contribution (without added salt)**								
**Beverage (alcoholic)**	**0.26** (0.00 to 0.54)	**0.09** (0.00 to 0.23)	**0.22** (0.00 to 0.49)	1.000<br>1.000<br>1.000	**0.06** (0.01 to 0.10)	**0.12** (0.00 to 0.23)	**0.02** (0.00 to 0.06)	1.000<br>0.809<br>1.000
**Beverage (non-alcoholic)**	**0.26** (0.03 to 0.49)	**0.07** (0.00 to 0.15)	**0.00** (0.00 to 0.00)	0.788<br>0.762<br>0.177	**1.86** (0.13 to 3.59)	**0.63** (0.00 to 1.39)	**0.71** (0.12 to 1.31)	1.000<br>1.000<br>1.000
**Bread and bakery products**	**0.93** (0.00 to 2.29)	**2.60** (0.14 to 5.07)	**1.30** (0.00 to 2.70)	1.000<br>1.000<br>1.000	**12.69** (7.58 to 17.79)	**13.33** (8.91 to 17.76)	**14.45** (8.80 to 20.10)	1.000<br>1.000<br>1.000
**Cereal, grains and products**	**4.74** (3.56 to 5.92)	**4.04** (2.87 to 5.21)	**4.14** (3.19 to 5.08)	1.000<br>1.000<br>1.000	**5.65** (3.16 to 8.14)	**6.86** (2.58 to 11.14)	**3.27** (1.91 to 4.63)	1.000<br>0.720<br>0.615
**Dairy and dairy products**	**35.15** (28.53 to 41.74)	**37.15** (32.10 to 42.21)	**41.74** (38.15 to 45.34)	1.000<br>0.896<br>0.533	**45.85** (38.10 to 53.60)	**48.52** (38.96 to 58.09)	**56.53** (48.75 to 64.32)	1.000<br>1.000<br>0.353
**Fats and edible oils**	**0.03** (0.00 to 0.08)	**0.00** (0.00 to 0.00)	**0.00** (0.00 to 0.00)	1.000<br>1.000<br>1.000	**1.21** (0.03 to 2.38)	**1.12** (0.09 to 2.14)	**0.72** (0.00 to 1.52)	1.000<br>1.000<br>1.000
**Fish and seafood**	**7.71** (3.71 to 11.72)	**5.99** (2.09 to 9.90)	**7.78** (6.07 to 9.48)	1.000<br>1.000<br>1.000	**0.14** (0.00 to 0.40)	**0.00** (0.00 to 0.00)	**0.06** (0.00 to 0.16)	1.000<br>1.000<br>1.000
**Fruits and vegetables**	**17.98** (13.58 to 22.38)	**15.72** (13.82 to 17.62)	**14.12** (11.83 to 16.41)	1.000<br>1.000<br>0.781	**19.96** (16.63 to 23.28)	**16.49** (14.14 to 18.84)	**16.77** (14.15 to 19.40)	0.585<br>1.000<br>0.860
**Meat, poultry and eggs**	**32.50** (23.03 to 41.98)	**34.06** (26.82 to 41.29)	**29.70** (25.87 to 33.53)	1.000<br>1.000<br>1.000	**5.31** (1.51 to 9.11)	**3.78** (2.06 to 5.50)	**2.58** (0.90 to 4.26)	1.000<br>1.000<br>1.000
**Snack foods**	**0.08** (0.00 to 0.25)	**0.00** (0.00 to 0.00)	**0.67** (0.00 to 2.15)	1.000<br>1.000<br>1.000	**7.23** (3.91 to 10.54)	**9.00** (5.29 to 12.71)	**4.83** (1.39 to 8.28)	1.000<br>0.654<br>1.000
**Sugar, honey and related products**	**0.35** (0.22 to 0.49)	**0.26** (0.20 to 0.33)	**0.32** (0.20 to 0.45)	1.000<br>1.000<br>1.000	**0.06** (0.00 to 0.15)	**0.15** (0.00 to 0.37)	**0.04** (0.00 to 0.08)	1.000<br>1.000<br>1.000

NOTE: first p-value reported is for the comparison between 20-39 and 40-59; second p-value is for the comparison between 40-59 and 60y up; third p-value is for the comparison between 20-39 and 60y up The analysis is based on regression (compatible with svy command). Post-estimation *contrast* command was used to compare the age group in each state and age group by state. The analysis is adjusted for multiple comparisons using Bonferroni's method.

Table S3: Mean percent salt contribution by area

Food group	Andhra Pradesh				Delhi and Haryana			
	Slum (n=251)	Urban (n=280)	Rural (n=226)	p-value	Slum (n=152)	Urban (n=126)	Rural (n=248)	p-value
**Added salt**	**88.97** (87.29 to 90.65)	**90.49** (88.25 to 92.72)	**86.44** (85.45 to 87.43)	1.000<br>0.009<br>0.073	**83.10** (80.88 to 85.32)	**80.60** (77.34 to 83.86)	**86.01** (84.63 to 87.40)	1.000<br>0.019<br>0.183
**Mean percent sodium contribution (without added salt)**								
**Beverage (alcoholic)**	**0.80** (0.26 to 1.35)	**0.10** (0.00 to 0.21)	**0.20** (0.00 to 0.46)	0.087<br>1.000<br>0.311	**0.03** (0.00 to 0.08)	**0.02** (0.00 to 0.05)	**0.12** (0.04 to 0.21)	1.000<br>0.165<br>0.384
**Beverage (non-alcoholic)**	**0.44** (0.00 to 0.94)	**0.24** (0.00 to 0.55)	**0.11** (0.00 to 0.27)	1.000<br>1.000<br>1.000	**0.29** (0.09 to 0.49)	**2.04** (0.00 to 4.36)	**0.95** (0.20 to 1.70)	0.873<br>1.000<br>0.583
**Bread and bakery products**	**0.00** (0.00 to 0.00)	**3.57** (0.99 to 6.16)	**0.70** (0.00 to 1.66)	0.046<br>0.261<br>0.915	**17.85** (13.13 to 22.58)	**16.77** (11.05 to 22.49)	**8.97** (4.92 to 13.02)	1.000<br>0.185<br>0.035
**Cereal, grains and products**	**4.59** (4.10 to 5.07)	**6.52** (5.66 to 7.39)	**3.59** (2.95 to 4.23)	0.001<br><0.001<br>0.097	**7.37** (4.76 to 9.99)	**7.93** (4.06 to 11.81)	**3.41** (2.04 to 4.78)	1.000<br>0.197<br>0.057
**Dairy and dairy products**	**30.06** (25.37 to 34.76)	**46.64** (41.67 to 51.61)	**33.03** (28.92 to 37.14)	<0.001<br><0.001<br>1.000	**33.17** (28.92 to 37.41)	**32.01** (26.50 to 37.52)	**65.03** (60.60 to 69.45)	1.000<br><0.001<br><0.001
**Fats and edible oils**	**0.22** (0.00 to 0.60)	**0.02** (0.00 to 0.05)	**0.00** (0.00 to 0.00)	1.000<br>0.891<br>1.000	**1.44** (0.13 to 2.74)	**2.09** (0.54 to 3.64)	**0.20** (0.00 to 0.45)	1.000<br>0.118<br>0.421
**Fish and seafood**	**2.73** (0.00 to 5.56)	**0.95** (0.00 to 2.12)	**10.23** (8.18 to 12.29)	1.000<br><0.001<br><0.001	**0.85** (0.00 to 2.34)	**0.01** (0.00 to 0.03)	**0.00** (0.00 to 0.00)	1.000<br>1.000<br>1.000
**Fruits and vegetables**	**19.00** (16.51 to 21.48)	**20.03** (16.01 to 24.04)	**15.27** (11.81 to 18.73)	1.000<br>0.484<br>0.529	**26.73** (23.65 to 29.81)	**21.60** (18.29 to 24.91)	**14.16** (11.72 to 16.59)	0.167<br>0.003<br><0.001
**Meat, poultry and eggs**	**41.85** (36.21 to 47.48)	**21.04** (17.00 to 25.09)	**36.56** (29.41 to 43.71)	<0.001<br>0.002<br>1.000	**5.95** (3.52 to 8.38)	**5.47** (1.75 to 9.19)	**3.35** (0.48 to 6.23)	1.000<br>1.000<br>1.000
**Snack foods**	**0.01** (0.00 to 0.02)	**0.50** (0.00 to 1.23)	**0.00** (0.00 to 0.00)	1.000<br>1.000<br>1.000	**6.30** (1.45 to 11.15)	**11.91** (7.19 to 16.64)	**3.77** (1.86 to 5.67)	0.639<br>0.013<br>1.000
**Sugar, honey and related products**	**0.30** (0.18 to 0.42)	**0.38** (0.29 to 0.47)	**0.30** (0.18 to 0.43)	1.000<br>1.000<br>1.000	**0.03** (0.00 to 0.06)	**0.14** (0.00 to 0.34)	**0.04** (0.01 to 0.07)	1.000<br>1.000<br>1.000

NOTE: first p-value reported is for the comparison between slum and urban; second p-value is for the comparison between urban and rural; third p-value is for the comparison between slum and rural
The analysis is based on regression (compatible with svy command). Post-estimation *contrast* command was used to compare the area classification in each state and area classification by state. The analysis is adjusted for multiple comparisons using Bonferroni's method.

Table S4: Mean percent salt contribution by education level

Food group	Andhra Pradesh				Delhi and Haryana			
	Primary level (n=528)	Secondary level (n=130)	Tertiary level (n=99)	p-value	Primary level (n=285)	Secondary level (n=125)	Tertiary level (n=99)	p-value
**Added salt**	87.17 (86.00 to 88.35)	88.30 (86.35 to 90.26)	89.01 (86.43 to 91.60)	1.000<br>1.000<br>0.848	85.04 (83.18 to 86.90)	83.45 (79.36 to 87.53)	80.36 (77.72 to 83.00)	1.000<br>1.000<br>0.033
**Mean percent sodium contribution (without added salt)**								
**Beverage (alcoholic)**	0.22 (0.02 to 0.43)	0.24 (0.00 to 0.53)	0.08 (0.01 to 0.15)	1.000<br>1.000<br>1.000	0.08 (0.00 to 0.16)	0.12 (0.05 to 0.18)	0.00 (0.00 to 0.01)	1.000<br>0.004<br>0.526
**Beverage (non-alcoholic)**	0.04 (0.00 to 0.09)	0.27 (0.00 to 0.64)	0.55 (0.00 to 1.18)	1.000<br>1.000<br>0.682	0.76 (0.19 to 1.32)	2.34 (0.00 to 5.59)	1.32 (0.00 to 2.96)	1.000<br>1.000<br>1.000
**Bread and bakery products**	1.04 (0.00 to 2.17)	1.94 (0.00 to .51)	2.47 (0.00 to 6.81)	1.000<br>1.000<br>1.000	9.90 (5.16 to 14.64)	14.03 (7.96 to 20.09)	17.36 (9.10 to 25.62)	1.000<br>1.000<br>0.784
**Cereal, grains and products**	4.20 (3.44 to 4.96)	5.41 (4.40 to 6.41)	3.92 (2.54 to 5.29)	0.029<br>0.003<br>1.000	4.43 (2.71 to 6.15)	6.16 (1.97 to 10.35)	6.82 (2.74 to 10.90)	1.000<br>1.000<br>1.000
**Dairy and dairy products**	36.62 (33.34 to 39.91)	36.29 (27.70 to 44.89)	37.27 (27.73 to 46.82)	1.000<br>1.000<br>1.000	56.12 (50.05 to 62.20)	45.70 (37.09 to 54.32)	37.01 (29.19 to 44.82)	0.136<br>0.733<br>0.001
**Fats and edible oils**	0.02 (0.00 to 0.06)	0.01 (0.00 to 0.01)	0.03 (0.00 to 0.09)	1.000<br>1.000<br>1.000	0.38 (0.00 to 0.78)	1.32 (0.06 to 2.57)	2.33 (0.00 to 4.87)	0.977<br>1.000<br>0.841
**Fish and seafood**	7.37 (4.37 to 10.36)	9.83 (6.51 to 13.15)	1.94 (0.48 to 3.40)	0.674<br><0.001<br>0.001	0.01 (0.00 to 0.03)	0.00 (0.00 to 0.00)	0.35 (0.00 to 0.98)	1.000<br>1.000<br>1.000
**Fruits and vegetables**	15.83 (13.87 to 17.79)	18.15 (14.34 to 21.95)	18.71 (10.80 to 26.63)	0.957<br>1.000<br>1.000	19.04 (15.66 to 22.41)	18.18 (14.51 to 21.85)	16.96 (13.20 to 20.72)	1.000<br>1.000<br>1.000
**Meat, poultry and eggs**	34.24 (29.65 to 38.82)	27.37 (16.98 to 37.76)	34.44 (18.40 to 50.48)	1.000<br>0.388<br>1.000	3.13 (1.64 to 4.62)	5.17 (1.45 to 8.88)	6.28 (1.38 to 11.18)	1.000<br>1.000<br>1.000
**Snack foods**	0.15 (0.00 to 0.46)	0.00 (0.00 to 0.00)	0.34 (0.00 to 0.99)	1.000<br>1.000<br>1.000	6.13 (3.88 to 8.39)	6.84 (2.95 to 10.73)	11.45 (5.29 to 17.71)	1.000<br>1.000<br>0.775
**Sugar, honey and related products**	0.27 (0.22 to 0.33)	0.49 (0.16 to 0.83)	0.25 (0.01 to 0.39)	1.000<br>0.807<br>1.000	0.02 (0.00 to 0.05)	0.15 (0.00 to 0.38)	0.12 (0.00 to 0.33)	1.000<br>1.000<br>1.000

NOTE: first p-value reported is for the comparison between primary and secondary level; second p-value is for the comparison between secondary and tertiary level; third p-value is for the comparison between primary and tertiary level. The analysis is based on regression (compatible with svy command). Post-estimation *contrast* command was used to compare the education level in each state. The analysis is adjusted for multiple comparisons using Bonferroni's method.

Table S5: Mean percent salt contribution by income in urban Andhra Pradesh

Food group	Middle income A (n=67)	Middle income B (n=73)	Upper income A (n=66)	Upper income B (n=74)	p-value
**Added salt**	89.49 (87.58 to 91.39)	92.21 (90.34 to 94.08)	89.09 (86.75 to 91.43)	88.74 (85.47 to 92.00)	All p's NS
**Mean percent sodium contribution (without added salt)**					
**Beverage (alcoholic)**	0.48 (0.00 to 0.95)	0.09 (0.00 to 0.28)	0.06 (0.01 to 0.10)	0.08 (0.00 to 0.18)	All p's NS
**Beverage (non-alcoholic)**	0.60 (0.00 to 1.27)	0.00 (0.00 to 0.00)	0.14 (0.00 to 0.31)	0.49 (0.00 to 1.00)	All p's NS
**Bread and bakery products**	0.33 (0.00 to 1.03)	2.96 (0.00 to 6.24)	2.17 (0.00 to 4.50)	4.67 (0.50 to 8.84)	All p's NS
**Cereal, grains and products**	4.34 (3.82 to 4.86)	7.63 (6.60 to 8.66)	3.69 (2.68 to 4.69)	5.68 (4.81 to 6.55)	MB vs MA: p<0.001<br>UA vs MB: p<0.001<br>UA vs UB: p=0.036
**Dairy and dairy products**	34.74 (29.69 to 39.78)	50.23 (45.96 to 54.51)	39.49 (28.10 to 50.87)	44.20 (34.76 to 53.64)	MB vs MA: p=0.001
**Fats and edible oils**	0.01 (0.00 to 0.02)	0.04 (0.00 to 0.09)	0.01 (0.00 to 0.02)	0.01 (0.00 to 0.01)	All p's NS
**Fish and seafood**	1.77 (0.00 to 3.84)	1.67 (0.00 to 3.64)	0.52 (0.04 to 1.00)	0.08 (0.00 to 0.24)	All p's NS
**Fruits and vegetables**	15.51 (11.51 to 19.50)	21.01 (13.28 to 28.73)	16.66 (15.12 to 18.21)	19.58 (16.35 to 22.82)	All p's NS
**Meat, poultry and eggs**	42.01 (33.05 to 50.97)	15.94 (11.00 to 20.88)	36.72 (25.15 to 48.30)	23.73 (19.99 to 27.48)	MB vs MA: p=0.002<br>UB vs MA: p=0.003<br>UA vs MB: p=0.030
**Snack foods**	0.01 (0.00 to 0.01)	0.00 (0.00 to 0.00)	0.00 (0.00 to 0.00)	1.14 (0.00 to 2.83)	All p's NS
**Sugar, honey and related products**	0.22 (0.14 to 0.30)	0.43 (0.35 to 0.50)	0.54 (0.06 to 1.02)	0.33 (0.19 to 0.47)	MB vs MA: p=0.023
